# From stressor to protector, UV-induced abiotic stress resistance

**DOI:** 10.1007/s43630-023-00441-1

**Published:** 2023-06-04

**Authors:** Gaia Crestani, Natalie Cunningham, Kristóf Csepregi, Uthman O. Badmus, Marcel A. K. Jansen

**Affiliations:** 1 School of Biological, Earth and Environmental Science and Environmental Research Institute, University College Cork, North Mall Campus, Cork, T23 TK30 Ireland; 2 Department of Plant Biology, Institute of Biology, University of Pécs, Ifjúság u. 6, Pécs, 7624 Hungary

**Keywords:** UV, UV-B, UV-priming, Drought, Necrosis, Abiotic stress, Cross-resistance acclimation, Climate change

## Abstract

Plants are continuously exposed to combinations of abiotic and biotic stressors. While much is known about responses to individual stressors, understanding of plant responses to combinations of stressors is limited. The effects of combined exposure to drought and UV radiation are particularly relevant in the context of climate change. In this study it was explored whether UV-exposure can be used as a tool to prime stress-resistance in plants grown under highly protected culture conditions. It was hypothesised that priming mint plantlets (*Mentha spicata* L.) with a low-dose of UV irradiance can alleviate the drought effect caused by a change in humidity upon transplanting. Plants were grown for 30 days on agar in sealed tissue culture containers. During this period, plants were exposed to ~ 0.22 W m^−2^ UV-B for 8 days, using either UV-blocking or UV- transmitting filters. Plants were then transplanted to soil and monitored for a further 7 days. It was found that non-UV exposed mint plants developed necrotic spots on leaves, following transfer to soil, but this was not the case for plants primed with UV. Results showed that UV induced stress resistance is associated with an increase in antioxidant capacity, as well as a decrease in leaf area. UV-induced stress resistance can be beneficial in a horticultural setting, where priming plants with UV-B can be used as a tool in the production of commercial crops.

## Introduction

Complex interactions between the stratospheric ozone layer and escalating changes in weather patterns, simultaneously affect the climate as well as the intensity of UV radiation reaching the Earth’s surface [1]. Historically, changes in UV irradiance have been strongly linked with the state of the stratospheric ozone layer, however, evidence is mounting that future alterations in UV irradiance will be more strongly associated with climate change effects namely altered cloud cover, surface reflectivity, aerosol concentrations, and solar energy penetration [2, 3]. Conversely, changes in stratospheric ozone can impact the climate, for example, the ‘hole’ in the Antarctic stratosphere is associated with changes in atmospheric air circulation and the local climate [1]. These interdependencies result in cascading effects that have potentially serious consequences for biodiversity, the economy, and human well-being [4]. Moreover, these interdependent effects expose plants to new combinations of UV radiation and other environmental change factors such as temperature and water availability. Alterations in availability of water, derived from a shift in precipitation patterns, cause heavy rains in some areas while other areas are affected by aridity [1]. In the latter case, water scarcity can potentially cause an alteration in plant growth and impact crop production and quality [5]. Furthermore, aridity caused by decreases in cloud cover will potentially be accompanied by increased local UV-irradiance, exposing plants to new combinations of enhanced UV and drought [1]. Areas that are predicted to experience more frequent dry periods include the Mediterranean, western North America, southwestern South America, South Africa, and southwestern Australia [6].

Plants, like other organisms, are continuously exposed to combinations of abiotic and biotic stressors [7]. While much is known about responses to individual stressors, understanding of plant responses to combinations of stressors is limited [8]. Furthermore, it appears that the response of plants to each combination of specific stressors is unique, making predictions of plant responses problematic [8]. In this context, the effects of combined exposure to drought and UV radiation are particularly relevant in relation with climate change [1]. Previous studies showed that both UV and drought have similar morpho-physiological effects in plants such as a reduction in leaf area and plant height, but increases in root biomass and leaf thickness [9–11]. For example Comont et al. [9] reported acclimatory responses including reductions in leaf area and specific leaf area in *Arabidopsis thaliana* plants after exposure to UV, drought and especially a combination of UV and drought. UV and drought exposures have also been associated with an increase in antioxidants, and metabolites such as flavonoids, terpenoids and carotenoids [10]. In particular, increased accumulation of the amino acid proline was reported, and this is associated with increases in plant stress resistance mediated through enhanced oxidative defence and plant signalling [12]. Proline metabolism under stress conditions is, in part, dependent on the hormone abscisic acid (ABA) [13]. Indeed, it is noted that both UV and drought interact with ABA metabolism, increasing ABA accumulation as part of a stress-response strategy [14]. An increase in ABA also mediates stomatal closure, decreasing transpiration under drought conditions [15]. The combination of different stressors may lead to the activation of multiple stress response-related pathways the effects of which may be additive, synergistic or antagonistic [7, 10]. A review of the relevant literature summarised that the simultaneous exposure of plants to drought and UV generates an acclimatory response that is not quite cumulative, while simultaneously negative stress effects are also less than cumulative [10]. For example, Cechin et al. [16] found that stomatal conductance was reduced both under UV and drought, although the reduction in plants exposed to combinations of the two stressors was not synergistic.

A special form of interaction between two stress-responses is when exposure to one stressor precedes that of a second stressor. It has been found that a UV pre-treatment can alleviate the effects caused by drought and vice versa, indicating a cross-resistance between the two factors [9]. UV-induced priming can potentially have a wide range of applications within the agricultural sector and can help plants harden and enhance stress resistance [17]. This specifically applies to plants pre-cultured in protected environments such as glasshouses. For example, Wargent et al. [18] showed that a pre-treatment with UV-B during the glasshouse growing stage of lettuce seedlings, resulted in higher yielding plants under field conditions. It was hypothesised that UV pre-treatment reduced the transplantation stress experienced when plants are transferred to outdoor conditions. An extreme example of a protected environment are in vitro conditions, whereby plants are raised under a high relative humidity, constant temperature, and an aseptic environment [19]. Upon transfer of in vitro plants to an ex vitro environment, plants are vulnerable to the drastic change in conditions, potentially affecting fitness and even survival [20]. In this study, it was explored whether UV-exposure can be used as a tool to prime plants grown under highly protected culture conditions, to induce a degree of stress resistance. It was hypothesised that priming mint plantlets (*Mentha spicata* L.) with a low-dose of UV irradiance can alleviate the drought effect caused by a change in humidity upon transplanting. It was also hypothesised that the increase in plant resistance would be accompanied by measurable changes in traits associated with plant defences, including stomatal opening, leaf area, and root development.

## Materials and methods

### Plant material and germination stage

Mint (*Mentha spicata L.*) seeds were commercially sourced (Moles Seeds Ltd., Stanway, U.K.), and sterilised as described in Crestani et al. [21]. Briefly, approximately 20 seeds per box were sown on half strength Murashige and Skoog medium without sucrose (MS) and grown for 30 days in plastic boxes (RA40 plastic micro boxes, Sac 0_2,_ Deize, Belgium). A total of 6 boxes were used for each treatment and each experimental replicate. Seedlings were grown under ~ 180 μmol m^–2^ s^–1^ PAR provided by a LED lamp (AP673L, Valoya, Finland), the output of which was measured with a PAR meter (PAR special sensor, Skye Instrument Ltd, Powys, United Kingdom). The day/night regime was 14 h light (7:00 to 21:00) and 10 h dark, at a temperature of 20 ± 2 °C and a relative humidity (RH) of ~ 98% inside the tissue culture boxes. Relative humidity and temperature were measured with a portable data logger (EL-USB-2, Lascar Electronics, Whiteparish, United Kingdom).

### UV–exposure stage

**Fig. 1:**
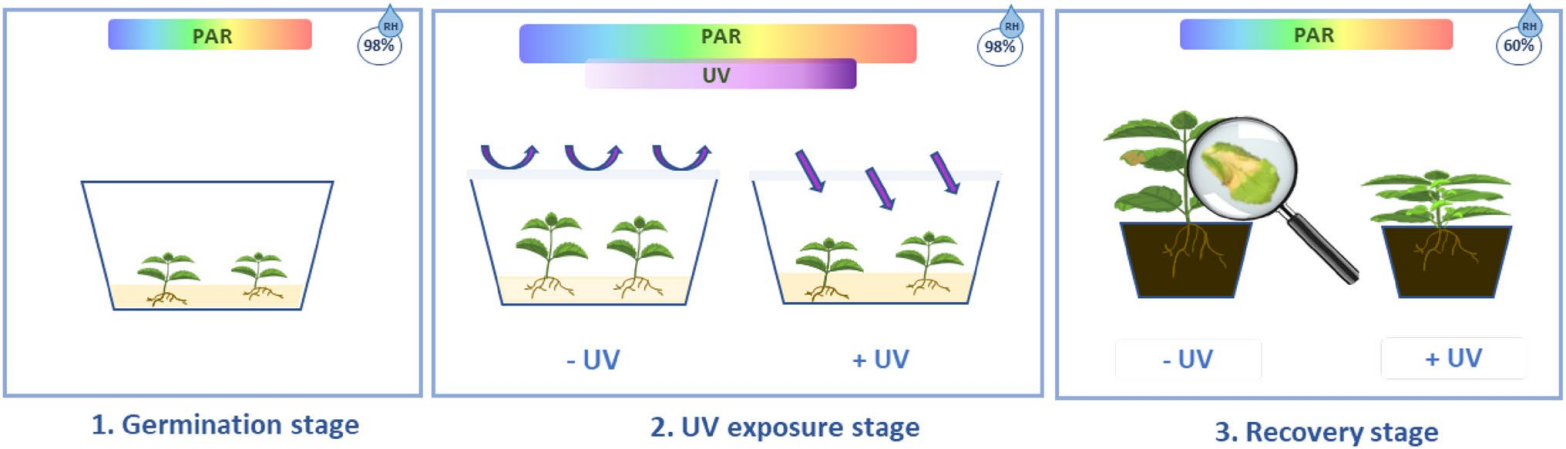
Plants were grown in tissue culture boxes for 30 days under PAR light (germination stage). Then, half of the plants were covered with UV-blocking filter and half with UV-transmitting filter and exposed for 8 days to UV and PAR (UV exposure stage). Finally, plants were transferred to soil for further 7 days (Recovery stage). Relative humidity inside the tissue culture boxes was around 98% while the relative humidity recorded during the recovery stage was around 60%

After 30 days of growth, seedlings were exposed to a UV treatment for eight days. Prior to UV treatment, the plastic containers were wrapped in either a UV-B blocking filter, Mylar (125 µm thickness, Polyester film, Tocana Ltd., Ballymount, Ireland) or a UV-B transmitting filter, Cellulose Acetate (CA) (95 μm thickness; Kunststoff-Folien-Vertrieb GmbH, Hamburg, Germany). Similarly, lids of containers were replaced by either a Mylar or CA cover. Filters were swabbed with 70% ethanol and replaced after 20 h of UV exposure (Fig. 1). UV-B radiation was provided by fluorescence tubes (TL40W/12 Philips, Germany) wrapped with a single layer of CA to block UV-C radiation. Plants were exposed to UV for 4 h at mid-day (12:00 to 16:00). Under the CA filter, plants were exposed to ~ 0.5886 W m^−2^ total UV (comprised of 0.3661 W m^−2^ UV-A and ~ 0.2225 W m^−2^ UV-B) and this treatment is labelled as “ + UV”. Under the Mylar filter, plants were exposed to 0.2498 W m^−2^ total UV (comprised of 0.2464 W m^−2^ UV-A and 0.004 W m^−2^ UV-B) and this treatment is labelled as “− UV”. The daily biologically effective UV-dose was equivalent to 2.7937 kJ m^–2^ (0.2830 kJ m^–2^ for UV-A and 2.5258 kJ m^–2^ for UV-B) under CA and 0.3250 kJ m^–2^ (0.1797 kJ m^–2^ for UV-A and 0.1453 kJ m^–2^ for UV-B) under Mylar [22]. Plants were exposed to ~ 180 μmol m^–2^ s^–1^ PAR as detailed for seed germination. PAR was supplemented by far-red LEDs tubes (L18C, Valoya, Finland) to achieve a red: far-red (R: FR) ratio of 1.6. The R: FR ratio was calculated as: photon irradiance between 655 and 665 nm/photon irradiance between 725 and 735 nm [23]. Both the UV irradiance and the R: FR ratio were mapped using an optical fiber spectroradiometer Flame-S equipped with a cosine corrector (Ocean Optics, Duiven, The Netherland) using the manufacturers software, Oceanview (version 1.6.7). All other growth room conditions were as specified for the seed germination stage.

### Recovery stage

Five plants per box were sampled immediately after eight days of UV exposure while the other five were transplanted on soil and monitored for further seven days (Fig. 1).The latter plants were transplanted on John Innes II compost (William Sinclair Horticulture Ltd., Lincoln, United Kingdom) with one plant per pot (7 × 7 × 8 cm, Walz, Effeltrich, Germany),and grown under the same conditions as reported for the germination stage but under the growth room relative humidity of ~ 60%. Potted plants were watered once during the recovery stage. Seven days after transplanting to soil, plants were harvested for analysis (Fig. 1).

### Necrotic areas

Leaf necrotic spots were noted on some plants at the end of the recovery phase. Necrotic areas (NA) were quantified from photographs of leaves using the software ImageJ (version 1.52a) (Wayne Rasband, National Institute of Health, United States). A necrotic spot was any dry and brownish area present on the adaxial leaf surface. The total leaf surface area covered by necrotic spots was quantified, and this was expressed as a ratio between necrotic area and total leaf area (NA/ LA). For all measurements, leaf one (L1) is defined as the oldest leaf and leaf six (L6) is defined as the youngest leaf. To assess the effect of the relative humidity on the formation of necrotic spots plants were exposed to different humidities during the recovery stage. After being exposed to UV, both non-UV and UV exposed plants were transplanted on soil and randomly divided in three groups, for exposure to low (~ 40%), normal (~ 60%), and high humidity (~ 90%). Plants were placed in seed propagator trays for the whole duration of the experiment. In order to define the humidity level and to monitor the humidity over time, a portable data logger was used.

Low humidity was obtained using silica pearls (drying pearl orange, Sigma Aldrich) that were spread on the bottom of a closed seed propagator tray and replaced every 48 h. High humidity was obtained by connecting the plant tray to a humidifier filled with distilled water. The extent of necrotic areas was recorded at the end of the recovery stage for three independent replicates, each comprising of six plants from each UV treatment. The evaluation was carried at the end of the recovery stage, on six plants per humidity level, and pre-treated or not with UV. There were three independent replicates.

### Proline content

Proline was extracted according to Alaiz et al. [24] with some modifications. A total of 100 mg of leaves was obtained by pooling together material from different plants and from different boxes, previously frozen in liquid nitrogen. Leaves were incubated for 24 h with 1 ml of 6 M HCl at 110 °C. Samples were filtered using 0.22 µm filters (Millipore, Bedford, USA). Extract (200 µl) was taken and evaporated under vacuum at 2000 rpm at 60 °C. Pellets were resuspended in 1 ml of 1 M borate buffer, and afterwards samples and standards were incubated with 2 µL of N,N′-Dimethylethylenediamine (DEEM) at 50 °C for 50 min. After incubation, 200 µL of the samples was transferred into HPLC vials. A calibration curve was constructed using different dilutions of a standard amino acid mixture (Merck Life Science Limited, Arklow, Ireland) at different concentrations, namely: 0.0125, 0.025, 0.0437, 0.625, 0.125, 0.1875 µM. The HPLC used was Agilent 1290 Infinity II LC (Agilent Technologies, Santa Clara, CA, USA) equipped with a column InfinityLab Poroshel 120 EC—C18, 2.1 × 50 mm, 1.9 µm (Agilent Technologies, Santa Clara, CA, USA), pressure 1300 bar, detector DAD UV 280 nm, autosampler temperature 10 °C, column compartment temperature 18 °C and 0.613 mL min^−1^ flow rate. The gradient program uses acetonitrile (solvent A) and sodium acetate 25 mM (solvent B) as  reported in Table 1.

**Table 1:** HPLC operating conditions used for the extraction of proline including the time (min), the gradient of solvent A (%) and solvent B (%), the flow (ml/min) and the maximum pressure (bar)

HPLC operating parameters				
Time [min]	A [%]	B [%]	Flow [ml/min]	Max pressure [bar]
0.30	12.00	88.00	0.613	1300
0.60	14.00	86.00	0.613	1300
1.30	14.00	86.00	0.613	1300
2.20	21.00	79.00	0.613	1300
3.50	31.00	69.00	0.613	1300
5.10	8.00	92.00	0.613	1300

Proline content was quantified after the UV exposure stage and again at the end of the recovery stage. Measurements were obtained from five independent experiments.

### Stomata

To quantify stomatal density and stomatal pore aperture on adaxial and abaxial surfaces, 12 leaves were picked randomly from amongst the first six (i.e., older) leaves of separate plants, collected from each UV treatment, either immediately after UV-exposure or after transplanting to soil. Additionally, stomatal density and pore aperture were also measured on leaves of plants before UV exposure. Six leaves were used to study the adaxial and abaxial leaf surface, respectively. For each leaf, measurements were taken at two locations, one near the leaf base and one near the leaf tip. Stomatal impressions were prepared following Wu & Zhao [25]. Leaves were detached from the plants and immediately painted with clear nail varnish. When dry, the leaf imprint was transferred on to a microscope slide, using a piece of clear tape. Stomata were identified and apertures were quantified using a Leica DM500 microscope (Leica Microsystems, Wetzlar, Germany) integrated with a camera with an objective magnification of 20 ×  and total magnification 200 × . Stomatal density and aperture were quantified in three different areas on each impression, each of which covered an area of 509 µm × 457 µm. The images were saved using Leica DM500 software (Leica Microsystems, Wetzlar, Germany) and then processed using ImageJ software (version 1.52a) (Wayne Rasband, National Institute of Health, United States). Stomatal opening was calculated as the distance between the two adjacent guard cells (width) [26]. A total of three independent replicates were carried out for each stage. The presence of some round microstructures between the guard cells in Fig. 7 represents an artefact of the tape used for making the epidermal impression.

### Antioxidant capacity

#### Extraction of plant material

Mint leaves were pooled together from different plants and from different boxes (25 mg of fresh weight), frozen in liquid nitrogen, were extracted using 300 µL of 70% ethanol, sonicated for 15 min in ice and centrifuged for 10 min at 3000 rpm. The pellet was resuspended 3 times and the supernatants were combined to give a final volume of 900 µL. The extract was used for Folin—Ciocalteau, ferric reducing antioxidant power (FRAP) and Trolox equivalent antioxidant capacity (TEAC) assays following Csepregi et al. [27] with some modifications. Antioxidants were measured after the UV exposure stage as well as after the recovery stage on five independent experiments.

#### Folin–Ciocalteau assay

An aliquot of 100 µL of ethanolic extract was mixed with 500 µL Folin–Ciocalteau reagent (1:10 dilution in distilled water). After 5 min at room temperature, 500 µL NaCO_3_ 6% (w/v) was added. After incubation for 1.5 h in the darkness, the antioxidant capacity was determined by measuring the absorbance at 765 nm using a UV–VIS spectrophotometer (Shimadzu UV—160 A, Kyoto Japan). A calibration curve was constructed using gallic acid as the standard and using different concentrations: 0.01, 0.05, 0.1, 0.25, 0.5 and 1 mg/ml. The results were reported in gallic acid equivalents.

#### Ferric-reducing antioxidant power (FRAP)

Ethanolic extract (50 µL) was mixed with 950 µL of FRAP solution and incubated for 30 min, mixing every 10 min, before measuring the absorbance with a UV–VIS spectrophotometer at 593 nm. The FRAP solution was obtained by mixing 12.5 ml of acetate buffer 300 mM (pH 3.6) with 1.25 ml of 2,4,6-Tris(2-pyridyl)-s-triazine (TPTZ) solution 10 mM in HCl 40 mM and 1.25 ml of FeCl_3_ 20 mM in distilled water. A calibration curve was made using different concentrations of ascorbic acid as the standard: 0.01, 0.05, 0.1, 0.25, 0.5 and 1 mg/ml. The results were reported as ascorbic acid equivalents.

#### Trolox equivalent antioxidant capacity (TEAC)

2,2′Azino-bis (3-ethylbenzothiazoline-6-sulfonic acid) cation radical (ABTS^·+^) solution was prepared by mixing 9.7 ml of 50 mM phosphate buffer (pH 6), 100 µl of 10 mM ABTS in 70% ethanol, 100 µl of 100 mM H_2_O_2_ in distilled water and 100 µl of 1.25 µM horseradish peroxidase in the phosphate buffer. After 15 min, 950 µL of ABTS^·+^ solution was mixed with 50 µL of the ethanolic extract and the absorbance was measured immediately at 730 nm using a spectrophotometer. A calibration curve was made using different concentrations of Trolox (6-hydroxy-2,5,7,8-tetramethylchroman-2-carboxylic acid) as standard: 0.00375, 0.0115, 0.0262, 0.0525, 0.105 mg/ml. The results were reported as Trolox equivalents.

#### Total UV-absorbing compounds

UV-absorbing compounds were measured following Csepregi & Hideg [28] with some modifications. A volume of 300 µl of ethanolic extract, the same used for the previous assays, was mixed with 1.5 mL of acidified ethanol (EtOH: H_2_O: HCl, 70:29:1) in a quartz cuvette. The absorbance was recorded in the UV range (280 nm–400 nm) with a spectrophotometer. The results were integrated across the whole wavelength range and the total UV absorbing compound concentration was calculated. Alternatively, absorbance was separately integrated across the UV-A (315 nm–400 nm) or UV-B (280 nm–315 nm) wavelength range. A calibration curve was made using different concentrations of quercetin as standard: 19.85, 39.70, 59.56, 79.41 and 99.26 µM.

### Chlorophyll a fluorescence analysis

Analysis of photosynthetic efficiency was performed using an Imaging-PAM Chlorophyll fluorometer with ImagingWin 2.47 software (Walz, Effeltrich, Germany). All samples were dark-adapted for 20 min, prior to the measurement of Fv/Fm. To measure the steady-state yield of photosystem II, and the quenching parameters, a background of 185 µmol m^−2^ s^−1^ of blue actinic light was applied for 5 min until a steady state was reached. Using a saturating pulse, Y(II) [29], Y(NPQ), and Y(NO) were then calculated [30]. Measurements were taken randomly on six healthy plants for each treatment from five independent experiments. For the plants that present necrotic areas, measurements were measured on the remaining, healthy section of the leaves. Chlorophyll *a* fluorescence measurements were performed on plants prior to UV exposure, at the end of eight days of UV exposure, and at the end of the recovery stage.

### Morphological parameters

Morphological parameters, including total leaf area, specific leaf area (SLA), fresh and dry root weight, and the length of the primary and secondary roots were measured as described in Crestani et al. [21]. In essence, the total leaf area was quantified using ImageJ software (version 1.52a) (Wayne Rasband, National Institute of Health, United States) across the oldest six leaves. SLA was calculated as a ratio between leaf area and leaf dry weight for the same leaves used to measure the leaf area. Root dry weight was measured after drying cleaned roots for 5 days at 60 °C. The length of the primary and secondary roots was calculated using SmartRoot, an extension of ImageJ. In addition, the root/leaf ratio was calculated as a ratio between root dry weight divided by the total leaf dry weight. All the morphological parameters were measured before UV exposure, at the end of the UV- exposure stage and at the end of the recovery stage. Total leaf area and root/leaf ratio data comprised the main stem leaves, although it was noted that small leaves had formed on branches of UV-exposed plants [see [21]] at the end of the recovery stage. All the morphological parameters were obtained from six plants after each stage, independently replicated three times, with the exception of total leaf area and SLA which were obtained from 5 independent replicates.

### Statistical analysis

Two tailed two-sample t-tests, or the correspondent nonparametric Mann–Whitney test, were used to establish significant differences between non-UV treated and UV-treated plants All statistical analyses were performed using the software IBM SPSS Statistic v28 (Armonk, New York, US).

## Results

*M. spicata* plants were grown in closed containers covered with either UV-blocking (Mylar) or UV-transmitting (CA) filters. After eight days of UV exposure, plants were transplanted to soil and monitored for a further seven days. Proline content, stomatal density and stomatal aperture, total antioxidant capacity, UV absorbing pigments, the efficiency of PSII and some morphological parameters were measured after the UV exposure stage and again after the recovery stage while necrotic areas were measured only after the recovery stage.

### Necrotic areas

**Fig. 2:**
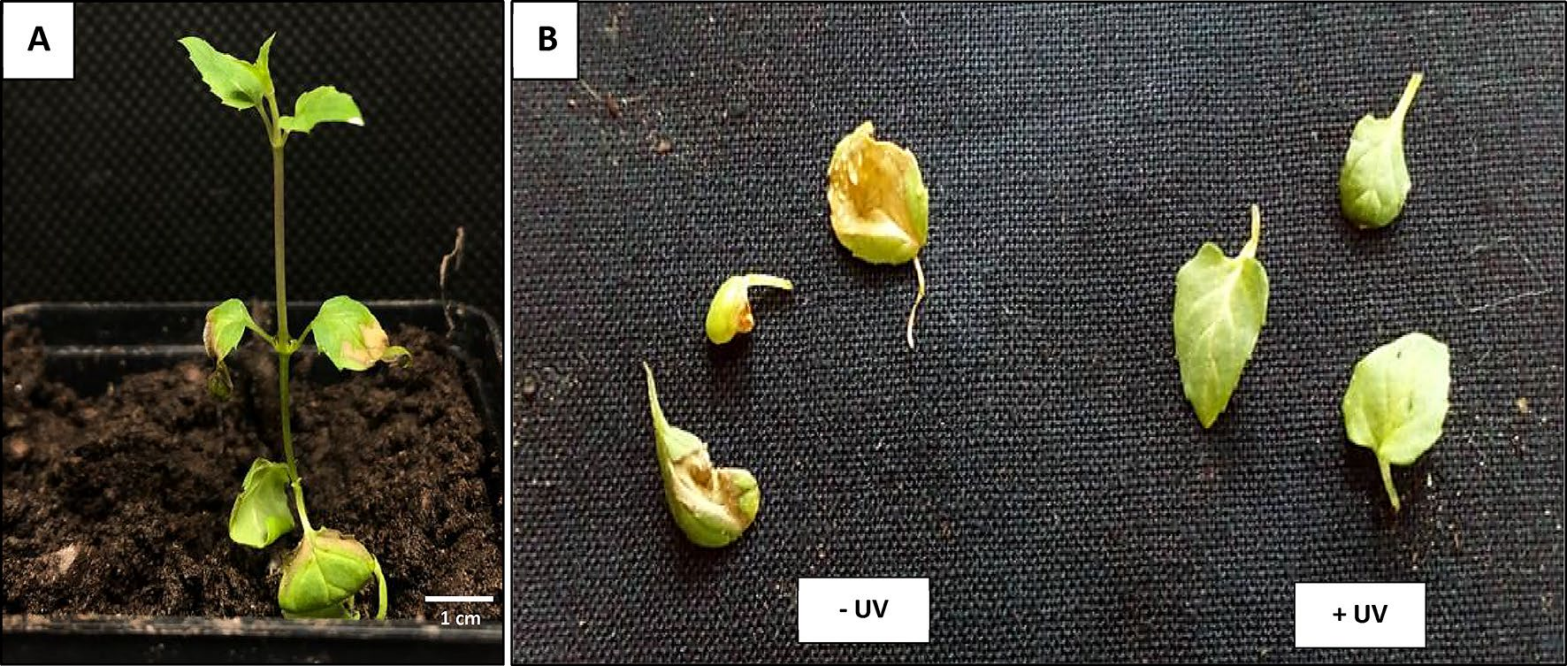
After the recovery stage, non-UV exposed plants show necroses on leaf surfaces (**A**). A comparison of non-UV exposed leaves (left) with UV exposed leaves, which do not present necrotic areas (**B**)

**Fig. 3:**
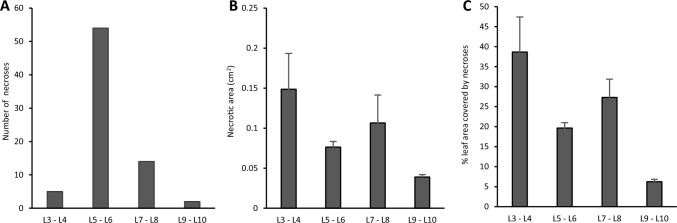
At the end of the recovery stage, 7 days after transplanting, necrotic areas were identified on non–UV exposed plants. Parameters measured were number of necroses across the leaves **(A)**, the dimension of the necrotic area (cm^2^) **(B)** and the % of the leaf area covered by necroses (**C**). Before UV exposure plants did not show any necroses**.** Also, UV-primed plants displayed no necroses

Necrotic spots occurred only after the recovery period and only in non-UV exposed plants (Fig. 2A). No necrotic spots were noted on plants that received a UV treatment (Fig. 2B). In contrast, 52% of non-UV exposed plants presented one or more necrotic areas. In total some 75 necrotic areas were identified across 75 leaves. These necrotic areas were found mainly on leaves five and six (L5–L6; 54 necrotic spots), followed by leaves seven and eight (L7–L8; 14 necrotic spots) and leaves three and four (L3–L4; 6 necrotic spots) and leaves nine and ten (L9–L10; 2 necrotic spots) (Fig. 3A). The average necrotic area was respectively 0.149 cm^2^ on L3–L4, 0.074 cm^2^ on L5–L6, 0.107 cm^2^ on L7–L8, and 0.039 cm^2^ on L9–L10 (Fig. 3B). The percentage of leaf surface damaged by necroses was calculated, and constituted 38.6% on L3–L4, 19.6% on L5–L6, 27.3% on L7–L8 and 6.2% on L9–L10 (Fig. 3C).

**Fig. 4:**
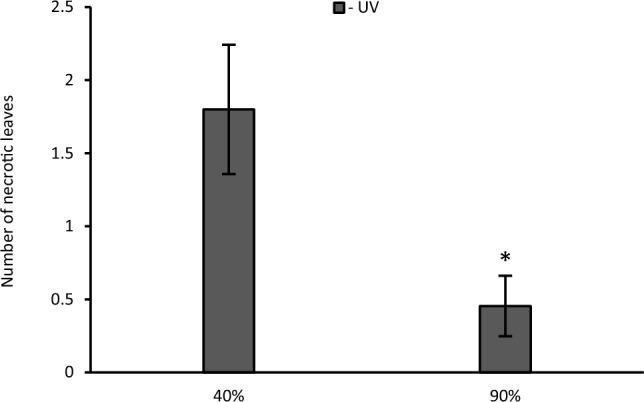
During the recovery stage, plants that did not receive UV (grey bar) were exposed to low (40%) and high (90%) humidity. After 7 days the mean number of necrotic leaves per plant was recorded. No necrotic spots were present in the UV pre-treated plants. Asterisks indicate a significant difference between the UV treatments with p < 0.05 (*)

To assess if the formation of necrotic spots was related to a change in the humidity, plants were exposed to 40% and 90% relative humidity (RH) during the recovery stage. A considerable number of damaged leaves were detected in plants exposed to 40% relative humidity. In comparison, the number of leaves with necroses was much higher on leaves from plants not exposed to UV at 40% humidity when compared to 90% humidity (*p* = 0.029). No necrotic spots were detected to plants pre-treated with UV (Fig. 4).

### Proline content

**Fig. 5:**
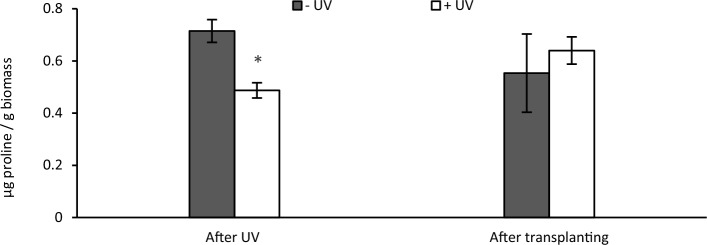
Proline content (μg proline/g biomass) was measured immediately after the 8 days UV exposure phase, or 7 days after transplanting. White bars UV-primed samples. Grey bars non-UV controls. Asterisks indicate a significant difference between the UV treatments with p < 0.05 (*)

The proline concentration was significantly higher in non-UV exposed plants than in UV-exposed plants after eight days of UV exposure (*p* = 0.008). A comparison after seven days recovery on soil showed that the proline concentration increased in previously UV- exposed plants, while it decreased in non-UV exposed plants (Fig. 5).

### Stomata

**Fig. 6:**
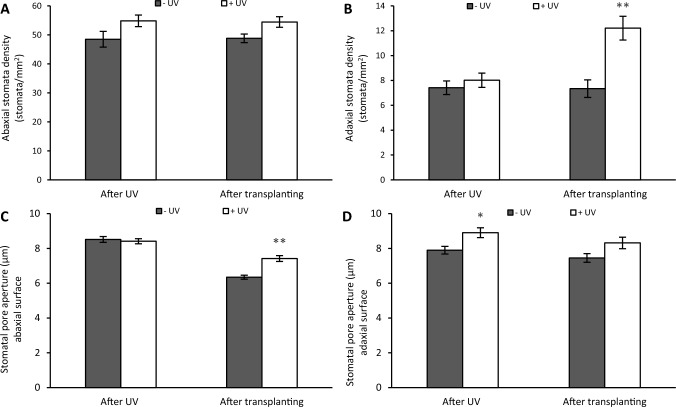
The stomatal density (stomata/mm^2^) on the abaxial (**A**) and adaxial surfaces (**B**), the stomatal pore aperture on the abaxial (**C**) and the adaxial surfaces (width) (**D**). Parameters were measured after the 8 days of UV exposure phase, and 7 days after transplanting. Before the UV exposure stage, the mean of the stomata density per mm^2^ was 43.87 on the abaxial surface and 7.63 on the adaxial surface, while the mean of the stomata pore aperture was 3.02 µm (width) on the abaxial surface and 2.95 µm (width) on the adaxial surface. White bars represent UV-primed samples while grey bars represent non-UV controls. Asterisks indicate a significant difference between the UV treatments with p < 0.05 (*) or p ≤ 0.001 (**)

**Fig. 7:**
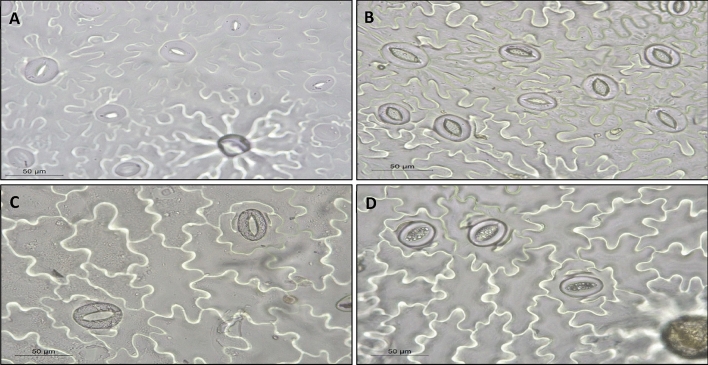
Stomata impressions at a magnification of 400X were obtained from the abaxial surface of non-UV exposed control plants (**A**), the abaxial surface of UV–exposed plants (**B**), the adaxial surface of non-UV exposed plants (**C**), the adaxial surface of UV–exposed plants (**D**) analysed at the end of the recovery stage

The stomatal density on the abaxial surface is not affected by UV, as measured either immediately after UV exposure or after recovery (Figs. 6A and 7A, B). Immediately after UV exposure, the stomatal density on the adaxial leaf surface was slightly, but not significantly, increased in UV-exposed plants. After the recovery stage, the stomatal density on the adaxial leaf surface of plants that had received UV was significantly increased, compared to non-UV exposed plants (*p* < 0.001) (Figs. 6B and 7C, D).

On the abaxial surface, the stomatal aperture was not immediately affected by UV treatment, but a significant difference was recorded after the recovery stage (*p* < 0.001). Stomata were more open in the UV-treated plants (Fig. 6C). On the adaxial surface, the stomatal pore aperture was greater in UV-exposed plants, but this difference was statistically significant only directly after the UV-exposure stage and not after the recovery stage (*p* = 0.006) (Fig. 6D).

### Antioxidants

**Fig. 8:**
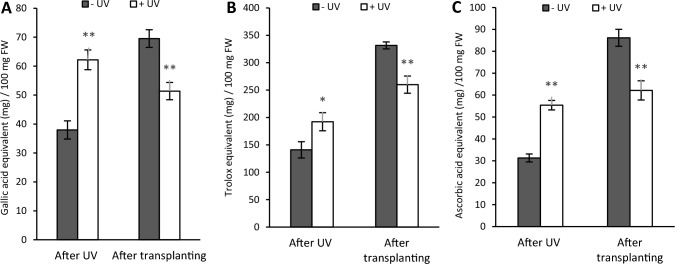
Total antioxidant capacity was measured as total phenols (gallic acid equivalent (mg)/100 mg FW) (**A**), as TEAC (Trolox equivalent (mg)/100 mg FW) (**B**) and as FRAP (ascorbic acid equivalent (mg)/100 mg FW) (**C**). Measurements were made at the end of the UV-exposure phase, and again at the end of the recovery phase. White bars UV-primed samples. Grey bars non-UV controls. Asterisks indicate a significant difference between the UV treatments with p < 0.05 (*) or p ≤ 0.001 (**)

The concentration of total antioxidants measured with the Folin-Ciocalteu assay increased in non-UV exposed plants over time. Immediately after UV exposure, UV has a positive effect on the total phenol concentration resulting in a significantly higher concentration in UV treated plants compared with the non-UV exposed plants (*p* < 0.001). However, this effect was temporary. After one week recovery, the total phenol concentration in previously UV-exposed plants was significantly reduced while in non-UV exposed plants it significantly increased (*p* < 0.001) (Fig. 8A). A similar trend was also observed for Trolox equivalent antioxidant capacity (TEAC). Immediately after the UV exposure stage, TEAC was significantly lower in non-UV-exposed compared to UV-exposed plants (*p* = 0.002). During the recovery stage, TEAC increased in both UV treatments, but the increases were far more marked in non-UV treated plants. As a result, TEAC was significantly higher in non-UV samples after the recovery stage (*p* < 0.001) (Fig. 8B). Similarly, immediately after UV exposure, ferric reducing antioxidant power (FRAP) is significantly higher in UV-exposed plants compared with the non-UV treated ones (*p* < 0.001). Following transfer to soil, FRAP values for non-UV exposed plants trebled, and as a result these plants have a significantly higher FRAP at the end of the recovery stage than UV exposed plants (*p* = 0.001) (Fig. 8C).

**Fig. 9:**
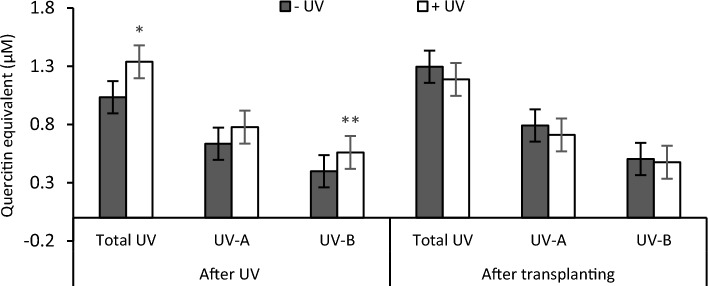
Total UV, UV-A and UV-B absorbing compounds were measured immediately after the UV exposure phase, and 7 days after transplanting. White bars UV-primed samples. Grey bars non-UV controls. Asterisks indicate a significant difference between the UV treatments with p < 0.05 (*) or p ≤ 0.001 (**)

Analysis of total UV absorbing compounds revealed a significant increase in UV-exposed plants measured immediately after the UV exposure phase (*p* = 0.019). This increase reflects the rise in UV-A and UV-B absorbing compounds but is significant only for UV-B screening pigments (*p* < 0.001). On the contrary, overall similar contents of total UV, UV-A, and UV-B absorbing pigments were recorded after the recovery stage (Fig. 9).

### Chlorophyll a fluorescence

**Fig. 10:**
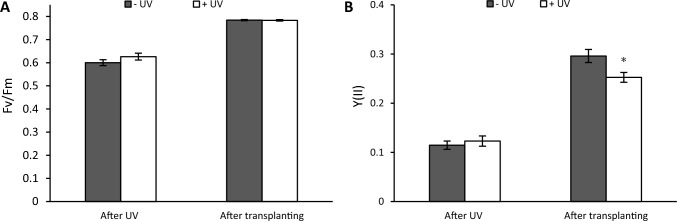
The maximum quantum yield of PSII, Fv/Fm (**A**) and the effective quantum of PSII, Y(II) (**B**) were measured immediately after the UV exposure phase and 7 days after transplanting. Before the UV exposure stage, the mean of Fv/Fm was 0.698 while the mean of Y(II) was 0.233. White bars UV-primed samples. Grey bars non-UV controls. Asterisks indicate a significant difference between the UV treatments with p < 0.05 (*)

To compare the impact of UV on photosynthetic efficiency, chlorophyll *a* fluorescence was analysed immediately after UV exposure and 7 days after transplanting on to soil. The maximum quantum yield of PSII, Fv/Fm, (Fig. 10A) was not affected by UV either, immediately after UV-exposure nor after the recovery stage. Also, the effective quantum of PSII, Y(II) showed no change between the two UV treatments at the end of the UV-exposure stage, but a significant reduction in Y(II) was noted in UV-exposed plants at the end of the recovery stage (*p* = 0.01) (Fig. 10B).

**Fig. 11:**
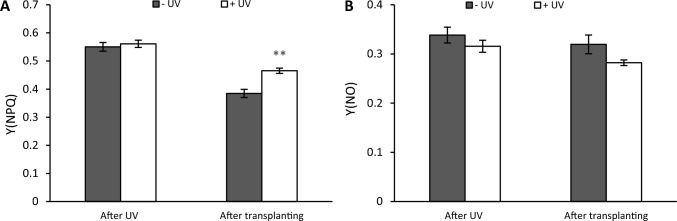
The energy dissipated through regulated non-photochemical quenching, Y(NPQ) (**A**), the quantum yield of non-regulated, non-photochemical energy dissipation Y(NO) (B) were measured both immediately after the UV exposure phase and 7 days after transplanting. Before the UV exposure stage, the mean value of Y(NPQ) was 0.478 while the mean value of Y(NO) was 0.287. White bars UV-primed samples. Grey bars non-UV controls. Asterisks indicate a significant difference between the UV treatments with p < 0.05 (*) or p ≤ 0.001 (**)

The energy dissipated through regulated, non-photochemical quenching, given by Y(NPQ) (Fig. 11A), showed no significant differences between UV treatments immediately after UV exposure. However, there was a significantly higher Y(NPQ) in UV-exposed plants after transplanting (*p* < 0.001). At the same time, the quantum yield of non-regulated, non-photochemical energy dissipation in PSII, Y(NO) (Fig. 11B), was not significantly affected by UV at either stage.

### Morphological parameters

UV-treated plants displayed a significant reduction in total leaf area, both immediately after UV-exposure (*p* < 0.001) and again after the recovery stage (*p* < 0.001), when compared with non-UV exposed plants. A substantial drop in SLA was recorded 7 days after transplanting, with considerably lower SLA values in UV-exposed plants compared to non-UV ones (*p* = 0.001). Fresh root weight was lower for UV-exposed plants after both experimental stages (*p* = 0.031 and *p* < 0.001). A non-significant difference in root dry weight was noted immediately after UV treatment, and this difference in root dry weight became significant seven days after transplanting to soil (*p* = 0.003) when the root biomass recorded for UV-exposed plants was lower than that for non-UV exposed plants. Primary and secondary roots were both significantly shorter in UV exposed plants in comparison with the non-UV exposed ones, after the UV- exposure stage (*p* = 0.011 and *p* = 0.006). Yet, no significant difference in primary and secondary root length was measured after the recovery stage. Finally, no difference between UV-treatments was found for the root/leaf ratio, both immediately after UV treatment and after the recovery stage (Table 2).

**Table 2:** Morphological parameters across different UV treatments, sampled immediately after the UV exposure phase or 7 days after transplanting on to soil

Parameter	− UV after exposure	+ UV afterexposure	Significance	− UV after transplanting	+ UV after transplanting	Significance
Total leaf area (cm^2^)	0.316 ± 0.01	0.250 ± 0.014	**	0.447 ± 0.045	0.270 ± 0.033 ^1^	**
SLA (cm^2^ mg^−1^)	0.51 ± 0.04	0.72 ± 0.12	n.s	0.37 ± 0.02	0.29 ± 0.02	**
Root fresh weight (mg)	16.9 ± 1.46	12.2 ± 1.4	*	26.7 ± 1.63	16.33 ± 1.25	**
Roots dry weight (mg)	0.99 ± 0.09	0.77 ± 0.05	n.s	2.41 ± 0.24	1.50 ± 0.17	*
Primary roots length (cm)	2.91 ± 0.24	2.25 ± 0.33	*	2.99 ± 0.55	2.97 ± 0.27	n.s
Secondary roots length (cm)	0.81 ± 0.06	0.69 ± 0.19	*	1.16 ± 0.37	0.70 ± 0.12	n.s
Root/leaf ratio	0.27 ± 0.05	0.23 ± 0.030	n.s	0.29 ± 0.028	0.28 ± 0.05 ^1^	n.s

Parameters include the total leaf area (cm^2^), SLA (cm^2^ mg^−1^), root fresh weight (mg), root dry weight (mg), primary roots length (cm), secondary roots length (cm) and root/leaf ratio. Before UV exposure, the mean of leaf area was 0.261 cm^2^, the mean of SLA was 0.448 cm^2^ mg^−1^, the mean root fresh weight was 8.00 mg, the mean root dry weight was 0.54 mg, the mean length of primary roots was 2.47 cm, the mean length of secondary roots was 0.80 cm and the mean root/leaf ratio was 0.184. Asterisks indicate a significant difference between the UV treatments with p < 0.05 (*) or p ≤ 0.001 (**) while non-significant results were identified as n.s

^1^Total leaf was measured only on the main stem leaves and not on the small leaves on newly emerging branches

## Discussion

In this study, it is shown that priming plants with UV enhances stress resistance. The data show that UV exposure generates more compact plants that cope better with the changes in environmental conditions.

### UV pre-treatment elicits stress resistance

Plants raised under in vitro conditions, in the absence of supplemental UV exposure, develop large numbers of necrotic spots once transplanted into soil. These necrotic spots are likely to be associated with the dramatic change in environmental conditions when a plant is transferred from a closed in vitro growth container to soil-based cultivation in a growth room. In vitro conditions include a high relative humidity, low levels of CO_2_ and a lack of wind exposure [31]. It was hypothesised that the dramatic decrease in RH, caused by transferring plantlets to growth-room conditions, is responsible for the development of abiotic stress in the in vitro raised plantlets, and the subsequent development of necrotic areas. Indeed, drought stress is often associated with formation of necrotic spots [32]. Moreover, by raising the RH to 90% necrosis was avoided. It was considered most likely that drought stress, due to an imbalance between water uptake and transpirational water loss, was the cause of necrosis. Remarkably, plants that had been raised under in vitro conditions, in the presence of supplemental UV, did not develop necrotic spots, once transferred to growth room conditions, not even under a low RH. Thus, it was hypothesised that UV induces drought resistance. These data are in agreement with a recent meta-analysis revealing a degree of cross-resistance between UV and drought [10]. Here, it is shown that UV-priming induces an array of acclimatory responses including morphological alterations, changes in stomatal density and aperture, changed proline content, and an elevated content of antioxidants and UV-absorbing pigments which may potentially contribute to drought resistance.

#### UV-induced changes in plant morphology

This study reports UV-induced decreases in total leaf area, as well as root length and weight. The results support previous findings wherein total leaf area decreased across different plant species including *Arabidopsis* [33], cucumber [34], and basil [35]. It has been theorised that these changes in leaf area are part of a defence response to minimize UV exposure and protect plants from UV stress [36], however, this has not been proven. Conversely, a decrease in leaf area is an effective response to limit transpirational water loss and increase drought resistance [37]. Thus, UV-induced changes in leaf area might be considered a key player in increasing the putative drought resistance reported in this study.

Interestingly, it was noted that UV negatively affects plant root systems with a decrease in both primary and secondary root length. This observation is consistent with earlier works. For example, UV inhibits root growth in UV-sensitive *Arabidopsis* mutants (*rus-1* and *rus-2*), [38, 39] a response that is mediated by the RUS1 and RUS2 genes [40]. Drought resistance is commonly associated with increased root development [41] which in turn leads to an improved water uptake capacity. For example, Jansen et al. [10] revealed a substantial increase in root: shoot ratio in plants exposed to drought. Thus, the data reported in this study do not support a role for UV-induced root formation in the plant resistance against abiotic stress. This particular morphological response needs to be investigated in more detail, as it may relate to the growth of plants on agar, and therefore be the result of direct UV exposure to roots.

#### UV-induced changes in stomatal responses

Under ideal growth conditions, plants lose more than 99% of water through stomatal transpiration [42]. Therefore, the question arises whether UV-induced changes in stomatal responses can explain the increase in putative drought resistance. Both the light spectrum and intensity are known to alter stomatal development and consequently their density as well as aperture [43]. Previous studies have reported that plants exposed to UV show a reduction in stomatal density. For example, Gitz III et al. [44], found a significant reduction of stomatal density in soybean, while Poulson et al. [45] reported a decrease in stomatal density in Douglas fir after exposure to ambient UV-B radiation. Conversely, Golob et al. [46] reported both UV-induced decreases as well as increases in stomatal density, depending on plant nutrition. In this study, both adaxial and abaxial stomatal density remained unchanged, despite UV exposure, and therefore it is concluded that this parameter does not explain any potential increase in drought resistance.

UV also modulates the stomatal aperture directly [26]. For example, Derebe et al. [47] reported substantial decreases in stomatal conductance in taro cultivars grown at different altitudes in Ethiopia. Indeed, many studies report that UV-B causes stomatal closure [48–50]. On the contrary, this study shows a slight UV-mediated increase in the stomatal aperture. Jansen & Van Den Noort [26] demonstrated that the UV effect on stomatal opening in *Vicia faba* plants depends on the metabolic state, with UV strengthening the background stomatal response. Consistent with this interpretation, it was observed that UV stimulates further stomatal opening under high humidity, and low CO_2_ conditions as found in in vitro containers. The data on enhanced stomatal apertures do, however, not explain the observed increase in drought resistance in mint plants.

#### UV-induced changes in cellular defences

Environmental stressors, such as UV-B and drought, can initiate signalling cascades that can induce biosynthesis of various stress-defence metabolites, including proline [51], antioxidants [52, 53] and UV-screening pigments [54]. Proline is an osmolyte that plants accumulate when exposed to environmental stressors such as salinity, high temperatures, drought and UV [10, 12]. Saradhi et al. [55] noted a time-dependent increase of proline across different plant species such as rice, mung bean and mustard during UV exposure. It was assumed that this increase indicated the involvement of proline in defence response against UV-generated radicals. Similarly, Salama et al. [56] found that UV increases the proline content of different varieties of desert plants such as *Plantago major, Malva parviflora, Rumex vesicarius, Sismbrium erysimoids* compared to the control. Although proline accumulation is commonly associated with stress defence, our data demonstrate that UV causes a small decrease in proline content, thus implying it is not responsible for any stress resistance in mint.

The antioxidant capacity of a plant cell can help protect against oxidative damage caused by a wide range of stressors [57]. Here, it is shown that UV exposure results in substantial increases in antioxidant activity measured as gallic acid, Trolox or ascorbate equivalents. In parallel, a strong increase in UV-screening capacity is measured. Induction of phenolic and flavonoid biosynthesis in UV-exposed plants may be regulated through the UV-B photoreceptor UVR8 [58]. The two acclimation responses are likely to be closely related to phenolics and other flavonoids playing a key role in increasing cellular antioxidant activities [59]. There is strong evidence that flavonoids and antioxidants also play a central role in protection against drought, both by preventing oxidative damage and through regulatory redox signalling [60]. Thus, it is concluded that the upregulated antioxidant defences in UV-exposed mint potentially contribute to improved stress resistance of the plants.

### The role of UV in stress -related parameters after a period of recovery

A strong acclimation response could be observed in both UV-primed and control plants during the recovery phase. Common acclimatory responses to drought are morpho-physiological re-adjustments including root expansion, stomatal closure, and accumulation of compatible solutes such as proline [61]. The data show an increased stomatal closure in non-UV exposed plants. This is most likely associated with drought stress, as stomatal closure is typically the first response of plants that experience drought [60]. A previous study showed increases in ABA in control plants during the recovery phase [21], and that is consistent with the observed stomatal closure [15].

Apart from minimising stomatal transpiration, both UV-primed and non-UV-primed plants increase their water uptake capacity through an increase in root biomass during the recovery stage. Secondary roots expand to increase the water uptake, thus minimising potential drought effects on plant growth [62]. The data in this paper show that plants are actively acclimating to the new, drier growth conditions.

In parallel, both UV-primed and control plants appear to minimise water loss by decreasing SLA. A reduction in leaf area versus leaf biomass is typically associated with drought acclimation [63]. Interestingly, although UV-primed plants did not show macroscopic sign of drought stress (i.e., necrosis) a particularly strong decrease in SLA is noticed. It appears that UV-primed plants are more effective in acclimating to drier conditions compared to non-UV- primed plants, suggesting that the substantial necrotic stress in the latter plants impeded acclimation. A likely explanation for the decrease in SLA is an increase in leaf thickness [64]. Leaf thickness tends to be positively associated with stomatal density [65], presumably to facilitate adequate CO_2_ supply with the increased mesophyll thickness. Consistently, in this paper, we noted that the lower SLA in UV-primed plants is associated with a higher stomatal density and aperture. The lack of increase in stomatal density in non-UV exposed plants is likely associated both with a higher SLA and in parallel with drought stress that occurs in these plants.

Overall, photosynthetic efficiency increased in all plants during the recovery phase. Acosta-Motos et al. [66] showed a progressive decrease in Y(NPQ) in *Stevia rebaudiana* over 28 days after the plants were moved to ex vitro conditions. This effect is likely to relate to an increase in CO_2_ availability, resulting in a lower protective Y(NPQ) but a higher Y(II) as was also observed in this study. Further, it could be speculated that UV-primed plants, not showing visible signs of UV stress and with higher stomatal gas exchange, will display a higher photosynthetic activity compared to plants not primed with UV. This is not the case. UV-primed plants display a lower Y(II) and a higher Y(NPQ) value, implying energy dissipation through the xanthophyll cycle [66]. Photosynthetic efficiency was measured in the non-necrotic regions of the leaf. Due to substantial necrotic leaf damage in control plants, there is less photosynthesising leaf area relative to root biomass in UV- primed plants. The imbalance will have increased the sink effect in plants not primed with UV. Such a sink effect is associated with a decrease in NPQ and an increase in overall photosynthetic efficiency [67]. Thus, a peculiar outcome of this study is that drought-stressed plants have locally a higher photosynthetic efficiency than UV-primed plants that display stress resistance, although this is likely to be a short-term affect that will not affect overall long-term plant photosynthetic productivity, and agro-technological applications.

The most common plant stress response is an increase in plant antioxidant defences [68]. Consistent with transplanting stress, a strong upregulation in antioxidant defence capacity is noted in all plants. This re-emphasises the conclusion that plants were actively acclimating. However, a stronger upregulation is noted in non-UV primed plants, consistent with the increased stress experienced by these plants. Interestingly, the higher antioxidant activity in non-UV primed plants is not associated with increased accumulation of total flavonoids and other UV-absorbing pigments, nor with proline accumulation. Rather, it is speculated that plants displaying drought stress will have accumulated other antioxidants, for example, ascorbate, glutathione, and tocopherol or have changed their flavonoid profile [69].

### UV-priming as a driving tool for sustainable crop production

In the context of a changing climate and consequential shifting plant production systems, there is a pressing need to explore novel ways to produce high-quality, cost-effective, hardened crops and ornamental plants. Drought stress is one of the most limiting factors in agricultural productivity, with estimated annual crop losses of 34% in the least developed and low to middle income countries [70]. In the current study, it has been theorised that UV-induced drought resistance and this can be beneficial in a horticultural setting. For instance, modern agriculture involves the transport of agricultural products worldwide and often involves unfavourable conditions including desiccating environments (e.g., airplanes, ferries, and trucks). Here, it is proposed that UV radiation can be used to produce hardened crops that can tolerate such sub-optimal growth conditions. UV induced drought resistance is associated with an increase in antioxidant capacity, as well as a decrease in leaf area. These responses are common amongst many crop species, and therefore it is speculated that priming plants with UV-B can be used in the production of many commercial crops as a means of enhancing resistance to negative environmental factors.
